# Evaluation of a new design solution for the visualisation of a risk-adjusted hospital performance comparison: results of an end user-centred mixed methods study

**DOI:** 10.1186/s12911-026-03501-5

**Published:** 2026-04-22

**Authors:** Niklaus S. Bernet, Michael Flückiger, Silvia Thomann, Jos M. G. A. Schols, Sabine Hahn, Leonie Roos, Mascha Kurpicz-Briki, Irma H. J. Everink

**Affiliations:** 1 School of Health Professions, Bern University of Applied Sciences, Applied Research & Development in Nursing, Murtenstrasse 10, Bern, 3008 Switzerland; 2 Institute of Design Research, Bern University of Applied Sciences, Bern Academy of the Arts, Fellerstrasse 11, Bern, 3027 Switzerland; 3 Department of Health Services Research, Care and Public Health Research Institute, Maastricht University, PO BOX 616, Maastricht, 6200 MD The Netherlands; 4 School of Engineering and Computer Science, Applied Machine Intelligence, Bern University of Applied Sciences, Höheweg 80, Biel, 2502 Switzerland

**Keywords:** Benchmarking, Inpatients, Decision making, Quality improvement, Quality of health care, Risk adjustment, Public reporting of healthcare data, Accidental falls, Pressure ulcer, User-centred design

## Abstract

**Background:**

Healthcare stakeholders are increasingly seeking comparative provider performance data to enhance data-driven decision-making and quality improvement. Traditional visualisations, like caterpillar plots, are often difficult for end users to understand and interpret. This study aimed to (1) obtain general feedback from end users on a newly proposed design solution for visualising a risk-adjusted hospital comparison and to develop an understanding of the key criteria they rely on in the evaluation process; (2) test the hypothesis that end users will better understand key messages and rate perceived usability higher with the new design solution than with a caterpillar plot.

**Methods:**

An end user-centred mixed methods study, involving end users of risk-adjusted hospital comparisons across all levels of the Swiss healthcare system, was conducted to evaluate the new design solution. In the qualitative phase, 14 end users from health authorities, insurers, hospital associations, and hospitals were surveyed in 10 semi-structured individual and group interviews, which were analysed using thematic analysis. In the quantitative phase, a non-clinical randomised controlled online trial (A/B testing) was conducted. In total, 200 of the targeted end users, comprising cantonal quality managers, hospital directors, and those responsible for quality and/or the ‘National Prevalence Measurement’ in hospitals, completed the questionnaire. The data were analysed using comparative descriptive and bivariate statistics.

**Results:**

Thematic analysis revealed three key criteria that end users relied on when evaluating a risk-adjusted hospital comparison: (1) ‘clarity by design’, highlighting strategies for effectively conveying key messages of hospital comparisons; (2) ‘usability by design’, focusing on end user-centred functionalities and presentation elements; (3) ‘suitability for quality development’, addressing the conditions for creating a trustworthy and useful comparison to drive quality improvement. Quantitative analysis confirmed the hypothesis that end users understand key messages better and perceived usability is higher with the new design than with the caterpillar plot.

**Conclusions:**

The new design solution improves hospital comparison outputs for end users by combining clear displays with additional interactive features. The identified criteria underlying the evaluation should inform further design projects and research dealing with the visualisation of hospital comparisons.

**Clinical trial number:**

Not applicable.

**Supplementary Information:**

The online version contains supplementary material available at 10.1186/s12911-026-03501-5.

## Background

**Fig. 1:**
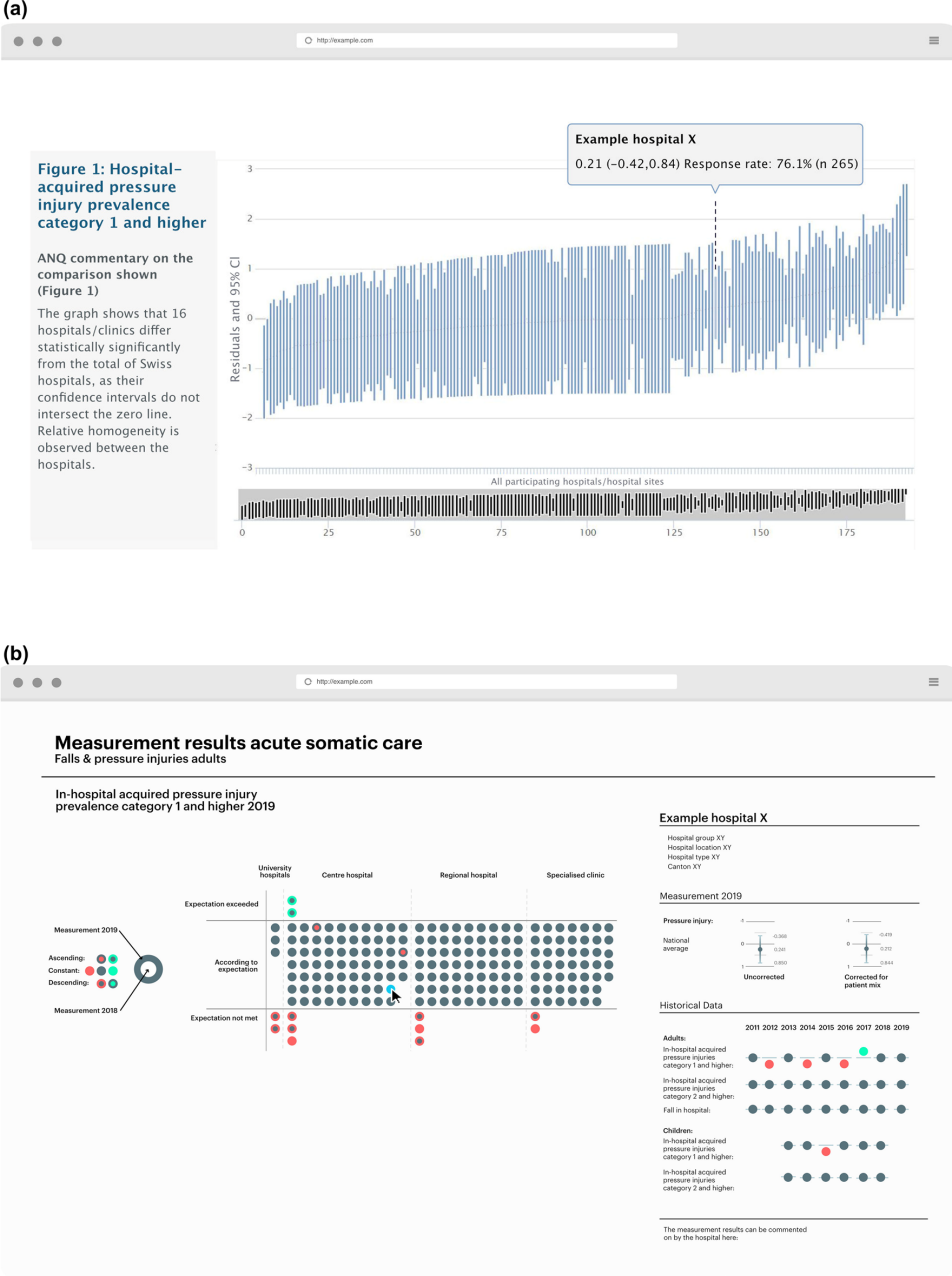
Visualisation of national hospital performance comparisons. (**a**) Translated example of a web-based interactive caterpillar plot for visualisation of national hospital performance comparisons, as publicly reported on the website (www.anq.ch*)* of the National Association for Quality Development in Hospitals and Clinics (ANQ) in Switzerland. Each bar in the plot represents the estimated hospital effect with the corresponding 95% confidence interval per hospital [6]. If the 95% confidence interval of the hospital effect does not intersect the zero line, it is assumed that this hospital deviates statistically significantly from the average of all hospitals and accordingly performs better (below the zero line [left]) or worse (above the zero line [right]) in relation to the outcome under investigation [7, 8]. (**b**) Mock-up presentation of the newly developed design solution for the visualisation of a risk-adjusted hospital performance comparison. The element on the left shows the overview display with the results for all hospitals and the element on the right shows the detailed display with the results for the hospital selected in the overview display

National quality measurements and the associated public release of performance data of healthcare providers are becoming common worldwide [1–3]. This public reporting is defined as ‘*the reporting of performance-related information to the general public about non-anonymous*,* identifiable professionals and providers (e.g.*,* individuals*,* institutions)*,* using systematically gathered comparative data*’ [1]. In contrast to the non-anonymous information on healthcare providers, the information on patients remains anonymous. The ultimate goal of public reporting is to drive quality improvement in healthcare by offering provider performance data, thus enabling a direct provider performance comparison, for example, in the form of a tabular or graphical non-anonymised hospital comparison [4, 5] (see Fig. 1a for an example from Switzerland).

The theory, based on the framework of Berwick et al. [9], describes two main mechanisms, known as pathways, on how public reporting contributes to improving quality of healthcare. In the ‘improvement through selection’ pathway, publicly reported provider performance data forms the basis for decision making by patients when selecting healthcare providers (healthcare professionals or organisations), but also by other organisations such as purchasers or regulators when it comes to contract negotiations or licensing. To avoid losing market share through poor results in provider performance comparisons or to gain new market share through positive results, healthcare providers are therefore motivated to introduce quality improvement measures [3, 9]. In the ‘improvement through change’ pathway, the provider’s performance data (also in comparison to others) is used to identify internal quality improvement potential and, triggered by the intrinsic motivation of healthcare professionals to provide the best possible quality, to introduce quality improvement measures [4, 9].

Therefore, various stakeholders such as governments, regulators, purchasers and healthcare providers, healthcare professionals and healthcare consumers are increasingly interested in accessing available comparative provider performance data to inform data-driven decision making [3]. However, comparative provider performance data can only realise its potential and stimulate quality improvement processes if the information provided is accessible, accurate and understandable to end users [2]. End users are defined as ‘*the person who is the ultimate recipient or user of a product*’ [10]. In this study, individuals are referred to as end users if it can be assumed (at least theoretically) that they could use provider performance comparisons (the product) as a source of information for decision-making processes in the context of their work or when selecting healthcare providers.

However, end users may find that such comparisons are not always presented in an easily understandable way. There is evidence that various end users, such as the general public, patients and healthcare professionals, sometimes experience difficulties in understanding, interpreting and using provider performance comparisons [11–13]. This challenge is particularly pronounced among those with limited data literacy or without formal training in data interpretation [14, 15]. As a result, visualisations like Fig. 1 may be difficult to interpret, especially when accompanied by limited or highly technical explanatory text.

The main reasons given for the limited understandability of provider performance comparisons are complex data visualisation approaches and presentation of information that is not tailored to the end users’ needs [16]. Accordingly, research over the last two decades has investigated and attempted to improve the understandability and usability of provider performance comparisons for end users such as the general public (including patients) [11, 12, 14–30], healthcare professionals [13, 24, 27, 31–37] and, to a limited extent, referring physicians [38] and legal decision makers [39].

Systematic reviews have yielded the following general design principles which, if taken into account in developing a new design solution, can contribute to improving both understandability and usability of provider performance comparisons for end users [16, 17, 24, 32]. First, simplify the performance data presented to reduce misinterpretation. Second, ensure uniform direction of the values presented (e.g., higher values equate to better performance). Third, use common and familiar colour schemes (e.g., red, yellow, green). Fourth, provide detailed information (e.g., confidence intervals) only when appropriate. Fifth, enrich complex data presentation approaches (e.g., caterpillar plots) with interpretation examples.

A further contribution to improving the usability of a new design solution is assumed if the expectations and needs of end users are already taken into account during its development [17, 40, 41]. Finally, it is crucial to evaluate a newly developed design solution within the end user target groups for which it was designed in order to verify the success of improvement measures and real-world usability [17, 42].

The founding of the National Association for Quality Development in Hospitals and Clinics (ANQ) in 2009 marked the beginning of a new era of public and transparent basic care hospital performance comparisons in Switzerland [43]. Since 2011, as part of the National Prevalence Measurement of Falls and Pressure Ulcers (NPM), the results of all acute care hospitals on the quality indicators of inpatient falls and pressure injuries have been published annually. These results are presented as a publicly accessible, non-anonymised graphical hospital performance comparison, which can be viewed on the website www.anq.ch as a national report or as an interactive graphic (Fig. 1a) [7, 44].

Although user feedback was not systematically recorded, anecdotal evidence from the helpdesk, which was available throughout the year to the hospitals participating in the NPM in Switzerland, suggested that some end users found the risk-adjusted hospital comparisons difficult to interpret. Risk adjustment is a key element in a fair and more accurate comparison of hospital performance, as it accounts for differences in the patient mix of providers [1, 7, 45]. However, as risk adjustment is typically based on complex, often multilevel, regression models, understanding the risk-adjusted results provided to the end user can be difficult without expert statistical knowledge [13, 25, 29, 46]. This was also reflected in the nature of helpdesk enquiries, which frequently concerned statistical aspects of the hospital performance comparisons, such as interpreting confidence intervals, identifying positive or negative outliers, and understanding a hospital’s ranking position. These challenges reinforce concerns, also reflected in the literature [32, 39], that the current approach of visualising risk-adjusted hospital performance comparisons using caterpillar plots, accompanied by limited explanatory text, may be difficult to understand or may even lead to misinterpretation by healthcare providers who are supposed to base their decisions on them.

To address the aforementioned issues and improve the correct understanding of the information conveyed by risk-adjusted hospital performance comparisons, new design solutions developed together with a wide range of end users would be highly desirable [47]. This is particularly important given that needs and expectations for the visualisation of a risk-adjusted hospital comparison may vary widely. Therefore, the so called vis(q)ual data project, a dual purpose project framed by action research according to Stringer [48], was launched in Switzerland in 2021 [49]. The overall project aims were: (1) to conduct a feasibility analysis to determine whether or not electronic medical record data from Swiss hospitals can be used for national quality measurement of inpatient falls and pressure injuries; (2) to collaborate with a broad range of end users in developing a new design solution to improve the understandability and usability of national risk-adjusted hospital performance comparisons and to evaluate this solution.

**Fig. 2:**
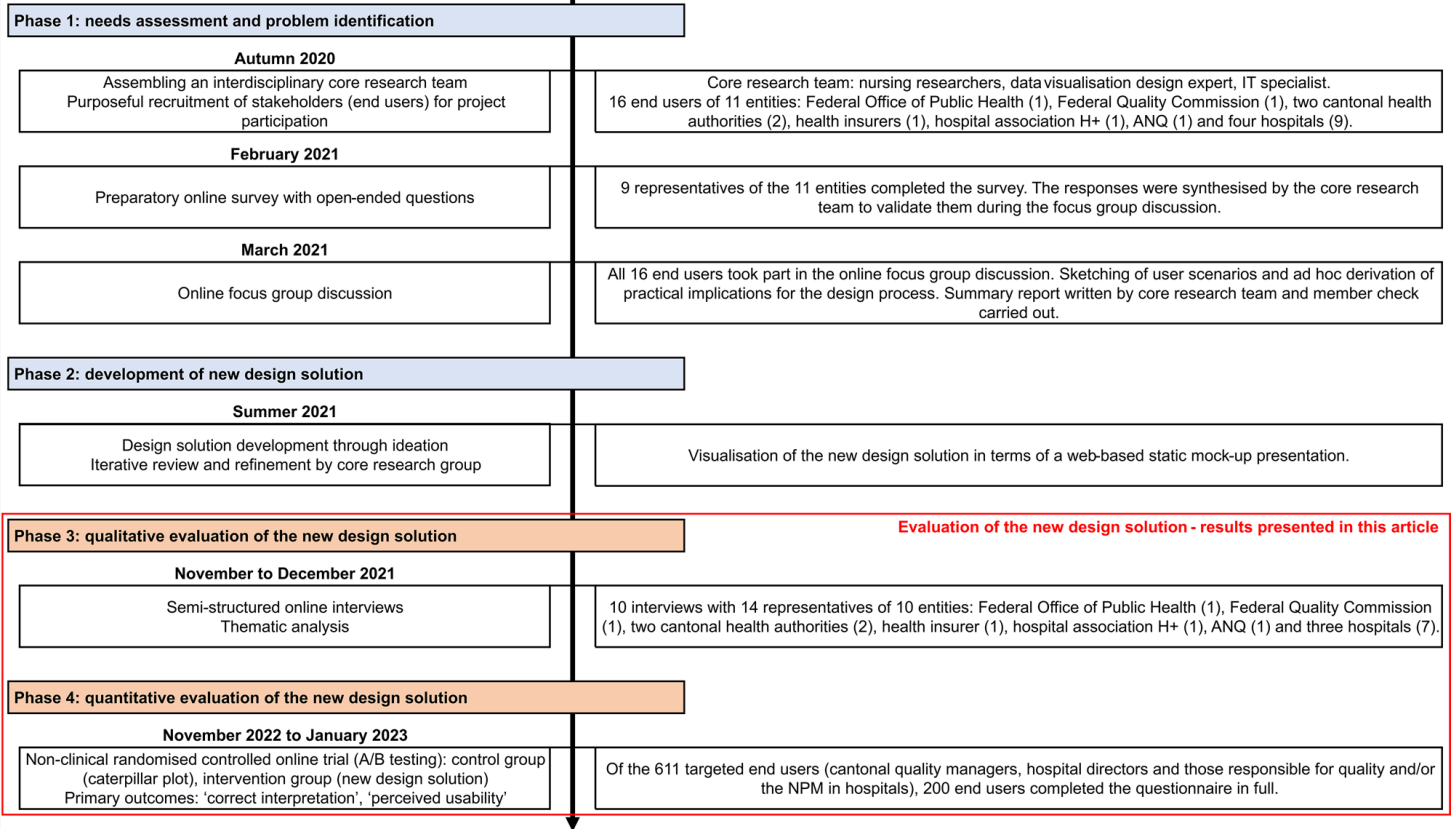
Description of the four phases with methods, analysed samples, and derived end products over time. ANQ, National Association for Quality Development in Hospitals and Clinics; NPM, National Prevalence Measurement of Falls and Pressure Ulcers

Results for the first aim are already published elsewhere [49]. Therefore, the present article focuses on the second project aim. The second project aim comprised four phases, as shown in Fig. 2.

In phases 1 and 2 a new design solution was developed by first surveying and then using a focus group to discuss the needs and expectations of 16 end users from 11 entities regarding risk-adjusted hospital comparison. Second, an iterative process was initiated under the direction of a design expert specialised in data visualisation in order to develop several design solutions through ideation. These were iteratively reviewed and refined within the interdisciplinary core research team (consisting of nursing researchers experienced in conducting national quality measurements and performing risk-adjusted hospital performance comparisons, an IT specialist and the design expert) until a final design solution emerged. The core elements of this solution were then visualised as a web-based static mock-up presentation (see Fig. 1b) based on the publicly available NPM 2018 and 2019 data [50, 51]. More detailed information on phases 1 and 2 can be found in the Additional file 1.

The new proposed design solution was then comprehensively assessed in phases 3 and 4 in a qualitative and quantitative evaluation with the end users. The aim of the qualitative evaluation was to obtain general feedback on the new design solution and to develop an understanding of the key criteria that they rely on when evaluating a new design solution for risk-adjusted hospital comparisons. The criteria underlying the evaluation contribute to science by providing insight into what requirements a meaningful hospital comparison must fulfil for end users and thus what criteria must be considered in future design processes related to meaningful risk-adjusted hospital comparisons. The aim of the quantitative evaluation was to test the hypothesis that end users will better understand key messages and rate perceived usability higher with the new design solution than with a caterpillar plot.

## Methods

### Study design

A user-centred, sequential mixed methods study design was used to evaluate the new design solution, which utilised both qualitative (phase 3) and quantitative (phase 4) methods (see Fig. 2). The qualitative evaluation consisted of semi-structured online interviews, conducted both individually and in groups with end users. These were analysed using thematic analysis according to Braun and Clarke [52] to identify relevant criteria relied upon by end users in the evaluation process. Thematic analysis is a structured, six-phase method for identifying patterns and themes within qualitative data. The six steps include: (1) data familiarisation, (2) generation of initial codes, (3) search for themes, (4) review of themes, (5) defining and naming themes, and (6) reporting [52]. The quantitative evaluation consisted of a non-clinical, between-subjects, randomised controlled online trial (A/B testing) [53]. In this trial, the primary outcomes, ‘correct interpretation’ of key messages and ‘perceived usability’ of a risk-adjusted hospital comparison visualised using the new mock-up design solution (intervention), were directly compared with the current caterpillar plot visualisation (control).

### Setting and sample

#### Qualitative evaluation

In Phase 3, the qualitative evaluation of the new design solution, we re-approached the same end users who had participated in phases 1 and 2. These participants had originally been recruited using purposive sampling to include individuals from different levels and functions within the Swiss healthcare system [54], based on the assumption that their needs and expectations regarding hospital performance comparisons would vary [1] (see Fig. 2 or Additional File 1 for a detailed description). However, one hospital, represented by two participants, could not be included in phase 3, as it withdrew from the study after completing phase 1 due to limited personnel resources.

Ultimately, 14 representatives from 10 different entities participated in the qualitative evaluation. These included one representative each from the Federal Office of Public Health and the Federal Quality Commission; two representatives from two cantonal health departments; one from a health insurer; one from the hospital association H+; one from ANQ; and seven representatives from three hospitals, including nursing experts, managers, and IT specialists.

#### Quantitative evaluation

A convenience sample was used for the quantitative evaluation. The aim was to include end users who were likely to have already come into contact with the visualisation of hospital comparisons based on caterpillar plots in the context of the NPM and, therefore, already had some prior knowledge. In the context of the Swiss healthcare system, it was assumed that individuals belonging to the following end user groups in particular fulfilled this inclusion criterion and were therefore defined as the target population: cantonal quality managers, hospital directors, hospital quality managers, and those responsible for executing the NPM in the hospitals. No additional inclusion or exclusion criteria were defined. In the comprehensive national Bern University of Applied Sciences (BFH) and ANQ contact databases, 611 individuals were identified as belonging to the target group. These individuals were contacted by ANQ via email with a request to complete the online questionnaire implemented in LimeSurvey (hosted at BFH). On the start page, participants were (again) informed about the background and purpose of the survey as well as the anonymous data collection and processing. By clicking on the survey link and proceeding with the survey participants gave informed consent to participate.

As web-based online surveys incur minimal incremental costs per additional participant, inviting the entire target population (*N* = 611) was feasible and allowed us to maximize representativeness without significant financial constraints. Therefore, no formal a priori sample size calculation was performed due to the census approach. However, participation was voluntary, and a total of 200 completed questionnaires (control group: 94; intervention group: 106) were available for analysis. To provide transparency regarding the precision and power of the findings, the 95% confidence interval for the difference in mean System Usability Scale (SUS) scores between groups is reported. A post-hoc power analysis using the website ClinCalc [55], based on the observed group sizes, means, and standard deviations, estimated approximately 88.7% power to detect the observed difference at a 5% significance level.

### Data collection

#### Qualitative evaluation

By means of semi-structured online individual and group interviews (via MS-Teams), the newly proposed design solution was discussed and evaluated with the end users. A total of ten interviews in Swiss German were scheduled, with one interview per institution. While eight interviews were held with a single representative (individual interviews), two were conducted as group interviews, each involving three representatives from the same institution. In the group interviews, the individual perspectives of the participants were asked and subsequently analysed separately.

An interview guide was created for the semi-structured individual and group interviews based on prior knowledge and the assumptions generated in phases 1 and 2. Following the interview guide, each interview started with a PowerPoint presentation by the core research team about the project results obtained so far. In particular, the steps taken, and the decisions made during the development of the new design solution were explained and visually illustrated. Subsequently, the following topics were discussed with the end users: general impression, ambiguities/understanding problems, problems with the current hospital comparison and the design solution, existing problems resolved with the new design solution, improvements/worsening, relevance/benefits of the design solution for end users and potential for further developments.

Led by an interdisciplinary team consisting of nursing researchers, a design expert, and an IT specialist, the individual and group interviews were conducted between November 22 and December 15, 2021, and lasted an average of 50 min (ranging from 40 to 70 min). The individual and group interviews were video-recorded, with participant(s) consent being obtained before the video recording began.

#### Quantitative evaluation

The data collected as part of the quantitative evaluation was gathered by means of an online survey conducted between November 16, 2022 and January 27, 2023. At the beginning, survey participants were randomly assigned to the control or intervention group with LimeSurvey automatically generating a random number (1 or 2) in the background. In the control group (1), participants were presented with the current visualisation in the form of a caterpillar plot and in the intervention group (2) with the newly developed design solution for evaluation. The images presented in Figs. 1a (control group) and 1b (intervention group) were used identically in the online survey. Although the survey did not explicitly inform participants whether they had been assigned to the control group (evaluating the current visualisation) or the intervention group (evaluating the new design solution), full blinding was unlikely. This is because we specifically targeted end users who were likely already familiar with the caterpillar plot visualisation used in the NPM context, making it probable that they recognised whether they were evaluating the current visualisation or the new design solution during the survey.

Both visualisations were presented in the survey as a web-based static mock-up presentation. Participants were then asked to answer a series of questions, which were identical for each group. These questions were compiled by the research team, drawing on similar studies [11, 19, 26, 38, 39]. The questions were divided into three parts. Part ‘A’ contained contextual questions about the end user. Part ‘B’ presented questions to assess the first primary outcome, the correct interpretation of the visual information presented. Finally, part ‘C’ used the well-known and psychometrically tested System Usability Scale (SUS) [56–58] to assess the second primary outcome, the perceived usability of the hospital comparison presented. Table 1 provides a complete overview of the outcomes and context variables that were collected and used in the quantitative evaluation. The Additional file 2 provides a detailed description of how the questionnaire was compiled, adapted and translated.

**Table 1:** Overview of the variables collected and used in the quantitative evaluation

Outcome variables	Coding	Remarks
**Correct interpretation (outliers)**		
Correct identification of the number of positive outliers	No [0]/ Yes [1]/ Don’t know [99]	If positively deviating hospitals were identified from the total of Swiss hospitals (= yes) and the number of positively deviating hospitals was correctly stated (= 2), the outcome variable was coded as yes (= 1).
Correct identification of the number of negative outliers	No [0]/ Yes [1]/ Don’t know [99]	If negatively deviating hospitals were identified from the total of Swiss hospitals (= yes) and the number of negatively deviating hospitals was correctly stated (= 14 [control]/10 [intervention]), the outcome variable was coded as yes (= 1).
Correct identification of inlier (average) hospital	No [0]/ Yes [1]/ Don’t know [99]	If the sample hospital was identified as an inlier hospital (being in line with the national average), the outcome variable was coded as yes (= 1).
**Composite Outcome**		
Correct identification of all positive, negative and inlier hospitals	No [0]/ Yes [1]/ Don’t know [99]	If the number of positive and negative outliers are identified correctly, as well as the example hospital as inlier, the composite outcome variable was coded as yes (= 1). If a case shows ‘don’t know’ on one of the three variables, this case is also coded as ‘don’t know’ (= 99) in the composite outcome variable.
**Correct interpretation (need for action)**		
Correct derivation of the need for action	No [0]/ Yes [1]/ Don’t know [99]	If no need for action was specified for the example inlier hospital (= no), the outcome variable was coded as yes (= 1).
**Perceived usability**		
SUS Score	0–100	The score was calculated according to Brooke [58] by converting the negatively worded SUS items 2/4/6/8/10 in such a way that, as with the remaining 5 items, a positive response represents a higher number. The sum of all 10 item values was then multiplied by a factor of 2.5 to reveal a score range from 0 to 100.
**Contextual variables**	**Coding**	**Remarks**
Swiss language region	German [1]/ French/Bilingual [2]/ Italian/Romansh [3]	Multiple answers were possible. Bilingual answers were only given for German-French. Bilinguals were coded into French. Romansh was combined with Italian.
Affiliation to hospital category	University hospital [1]/ Centre hospital [2]/ Regional hospital [3]/ Specialised clinic [4]/ Other organisation/Don’t know [5]	Hospital category defined by the Swiss Federal Office of Statistics [59]. The response categories ‘Other organisation’ and ‘Don’t know’ were combined.
Approximate role category in the organisation	Hospital Director/Nursing Director [1]/ (Head of) quality management [2]/ Nursing expert [3]/ Responsible for NPM [4]	The information entered in the text field was standardised and then divided into broad types of roles in the organisations (see column ‘Coding’).
Frequency of participation in the NPM (proxy measure for contact with hospital comparisons)	Once [1]/ Twice [2]/ ≥ 3 times [3]/ Don’t know [4]	

NPM, National Prevalence Measurement of Falls and Pressure Ulcers; SUS, System Usability Scale

### Data analysis

#### Qualitative evaluation

To evaluate the new design solution and deepen understanding of the key criteria that end users rely on in their evaluation, the interviews were analysed using a thematic analysis approach, following the six phases outlined by Braun and Clarke [52].

In the first phase (data familiarisation), the videos were viewed several times independently by two researchers (NB, LB). To separate the passages of the interviews that were relevant to the research question from the irrelevant content (e.g., pure presentation of previous project results), potentially important sections (guided by prior knowledge and assumptions from phases 1 and 2 of the project) were recorded by noting the respective time points of the videos. Once the relevant passages had been jointly clarified, they were all transcribed verbatim into standard German by a researcher (LB), ensuring the surrounding context was also transcribed to avoid taking passages out of context. The transcribed text passages were compiled in an Excel sheet at participant level, as suggested by Bree and Gallagher [60], and cross-checked with the videos by another researcher (NB). In the second phase (generation of initial codes), a deductive approach was initially chosen for the thematic analysis, which was based on the objectives and prior knowledge from phases 1 and 2 of the project. These predefined codes were then supplemented by inductively generated codes that emerged directly from the data. This process was carried out independently by both researchers (NB, LB). The derived codes were iteratively compared and modified. In the third phase (search for themes), similar codes were organised and grouped together by the two researchers independently in order to generate initial ideas of broader preliminary themes. In the fourth phase (review of themes), the preliminary themes were compared and a consensus was sought. The resulting thematic structure was visualised in the form of a mind map to review uniqueness of themes and highlight possible connections and relationships between the themes and sub-themes. In the fifth phase (defining and naming themes), the ‘essence’ of the final three themes with corresponding sub-themes was described and evaluated by the research team. In the sixth phase (reporting), the results of the analysis were presented together with corresponding quotes from end users. For publication, the original quotes were translated into English and subsequently reviewed by a native speaker and professional editor to ensure accuracy and clarity. In addition, an experienced researcher (CG) specialising in thematic analysis supervised and verified the entire coding process and the identification, collation and description of themes.

#### Quantitative evaluation

The survey results were analysed using standard descriptive statistics. The Pearson chi-square test and the t-test were used to compare results regarding the contextual information and the outcomes between the control and intervention groups. As only fully completed questionnaires were included in the analysis, there were largely no missing values in the data. The analyses were performed with IBM SPSS (version 29). A p-value of less than 0.05 was defined as the statistical significance level.

## Results

### Qualitative evaluation

The general feedback from all 14 participants indicates that the end users were enthusiastic about the new design solution. From the discussion, it can generally be concluded that, in end users’ view, the new design solution shows significant improvement over the current visualisation with caterpillar plots, as highlighted by the following selected quotes: *‘that’s what I always wished for and it [new design solution] actually contains everything.’* [I-2]; *‘a graphic like the one you have now presented here [new design solution] is much easier to understand and much clearer than the previous presentation [caterpillar plot].’* [I-3]; *‘I find it very aesthetic and much calmer for the eye*,* which has a very great added value.’* [I-4]; *‘So from my point of view*,* I would almost want to use a word like congratulations.’* [I-10].

**Fig. 3:**
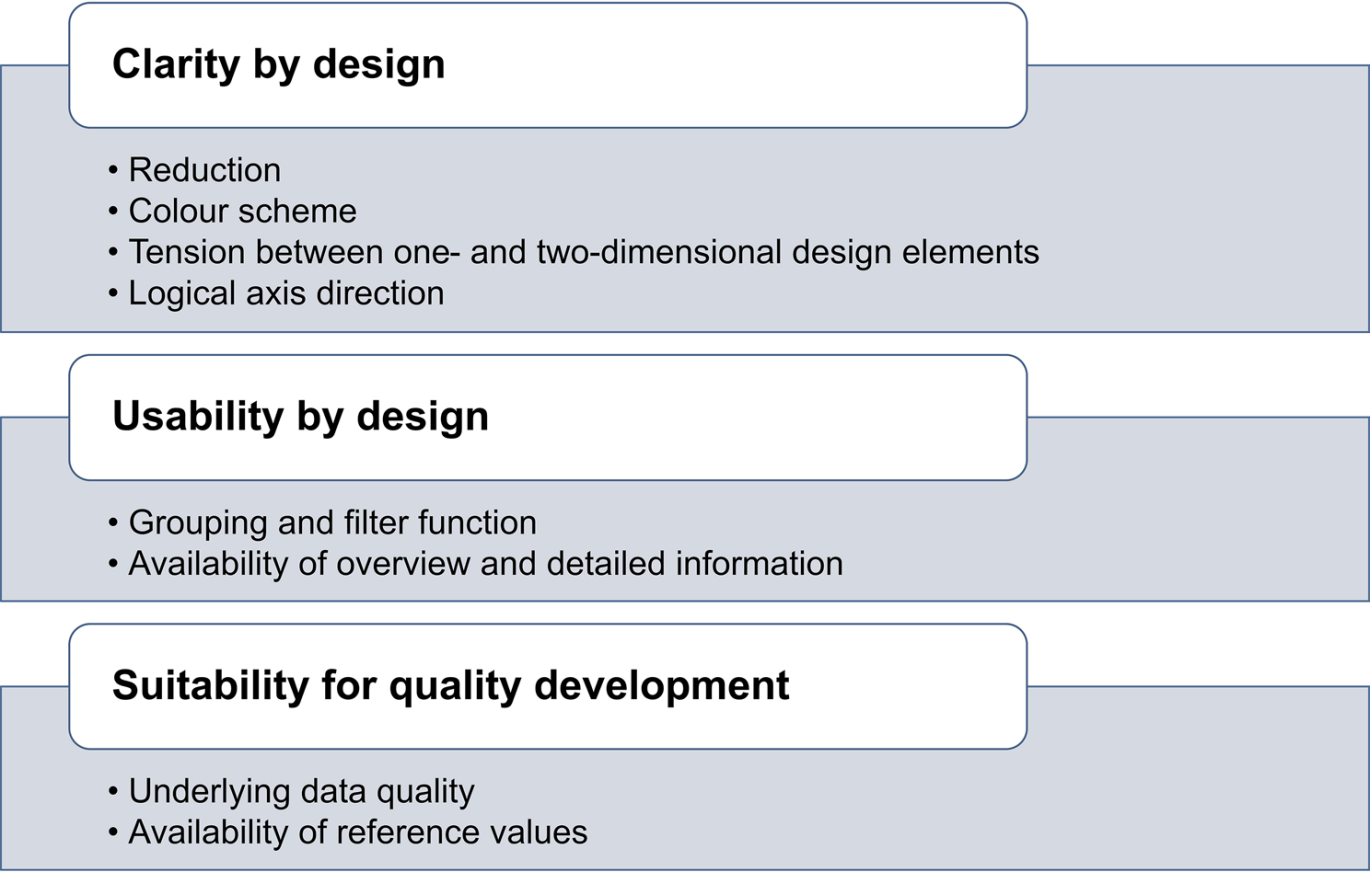
Overview of the key criteria and corresponding sub-themes identified in the thematic analysis

The thematic analysis yielded three main themes utilised by the end users as criteria in evaluating the new design solution: (1) ‘clarity by design’; (2) ‘usability by design’; (3) ‘suitability for quality development’. The three themes and corresponding sub-themes are summarised in Fig. 3.

### Clarity by design

All participants emphasised that the information to be conveyed by the hospital comparison should be visualised as clearly as possible. The clarity by design of a hospital comparison emerged as a key evaluation criterion for end users. The sub-themes described below illustrate how adjustments/decisions regarding the design elements, that is, the elements used in the presentation of the hospital comparison, positively or negatively influence the clarity of the information to be conveyed and thus the clarity of the hospital comparison.

#### Reduction

Deliberate omission of irrelevant, potentially misleading information in the sense of reduction can improve the clarity of hospital comparison. In the interviews, for example, it became clear that the rankings based on the hospital effect and their 95% confidence intervals, which are shown in the caterpillar plot for each hospital, are often misconstrued by end users, who identify the vertical blue line representing their hospital of interest and compare its position to that of other hospitals to the right or left. However, no such ranking is possible with this caterpillar plot, which actually only conveys three messages about a given hospital: significantly better than average, average, and significantly worse than average, indicated by whether (or not) a vertical (hospital) line crosses the 0 line (national average).

The removal of the hospital effects and their confidence intervals in the new design solution led to an improved and clearer mediation of the three intended messages to be conveyed in the overview display: the hospital deviates negatively (negative outlier), lies within the national average (inlier) or deviates positively (positive outlier) from the national average.

*Are we in the average range or are we just below or above it. That’s actually the only thing that’s really reliable. And the whole*,* I’ll say it a bit nastily*,* ‘hype’ about how far we are in the rankings*,* that falls away a bit*,* because the message is simply that we are average.* [I-9-2]

At the same time, however, it also became clear that although the reduction is generally welcomed for a quick overview and correct interpretation of the main messages, the 95% confidence intervals represent important information for some end users, especially experts with statistical knowledge. This underlines the benefit of providing such information elsewhere as additional information.

*I think it’s good that you (…) continue to make the detailed data with the residuals [hospital effects] available for the interested reader. Because for me*,* the residuals are very important and very interesting (…) but of course I am more of an expert and not a layman*,* so I would definitely leave them available. They may be in the background or listed separately.* [I-3]

Clarity by design is therefore not only a question of reducing information, but also of how the information provided is organised.

#### Colour scheme

In the evaluation, end users assessed the extent to which the colour scheme chosen, apart from personal preference, is suitable for highlighting and clarifying the visual information. The green-grey-red colour scheme proposed in the design solution was perceived as a significant improvement in terms of clarity over the monochrome caterpillar plot, which does not highlight outlier hospitals in any colour.

*What I definitely like is that you can see by colour*,* for example*,* whether a hospital shows a positive or negative deviation*,* in the sense of what is good or bad for the patient.* [I-7]

To further enhance the clarity of the design solution, the familiar traffic light colour scheme (green-yellow/orange-red) was suggested for highlighting positive/negative outliers.

#### Tension between one- and two-dimensional design elements

The evaluation revealed a tension between the need to enrich a hospital comparison with additional information and to improve clarity through reduction. The decisions made in the design process regarding the provision of one- or two-dimensional design elements appeared to be a relevant factor in influencing how much the visualisation was assessed as clear by the end users. This is reflected by the two-dimensional design element presented in the overview display of the new design solution. Using the colour scheme introduced, the historical positioning of a hospital (from outlier to inlier) is visualised by a dot, and the current positioning by a circle around the dot. By visually combining the historical and current positioning of a hospital, information on hospital-specific development over time is thus added to the overview display. This approach leads to condensation of the information to be conveyed, but also to greater complexity in the end user’s task of interpreting the information provided. Reduction and the colour scheme as measures chosen to improve clarity are therefore hampered by the provision of an additional layer of information in the two-dimensional design element.

*The rings you showed there*,* yes*,* you have to get used to them*,* the eye needs some time (…) It’s certainly exciting to mix historical and current data*,* but I still need a second look (…).* [I-3]

However, this additional information is also highly appreciated, especially by regulators and quality managers, as they would otherwise have to search for it manually. Some end users even requested the integration of further circles in order to display additional historical data.

#### Logical axis direction

Ensuring logical axis direction is a relevant criterion that contributes to the clarity of the messages to be conveyed by a hospital comparison. For example, in relation to the caterpillar plot, end users have criticised that the negative outliers (hospitals with more falls or pressure injuries after risk adjustment) are shown in the top right-hand corner, which runs counter to intuitive reading. The reversal of the axis direction in the design solution was assessed as contributing to clarity of the hospital comparison.

*I also think it’s good that you turn it around (…) because it’s really the case that you have a different association in your head.* [I-6]

### Usability by design

In order for a hospital comparison to provide diverse users with the necessary information according to their specific needs, it became apparent that the design features and elements must allow a certain degree of interactivity with the end users. A key evaluation criterion was therefore the envisaged ability of the new design solution to provide meaningful, user-oriented grouping and filtering options, as well as displaying additional detailed information.

#### Grouping and filter function

The decision taken during the design process to present individual hospitals in a matrix in the overview display allows hospitals to be arranged in meaningful groups and filtered according to various factors. End users value this interactivity and the ability to adapt the visualisation to their needs.

*(…) having several clusters provides completely new ways of looking at things compared to what we have at the moment. So I very much welcome that.* [I-4]

In particular, as it is assumed that these functions will reduce the additional work that was previously necessary, for example, when the results of the hospitals in a particular region (e.g., canton) had to be compiled manually. In order to meet the needs of the various end users of a hospital comparison – from patients and hospitals to insurers and cantons – the end users suggested including the following grouping/filtering options: distance from place of residence, department types, hospital size, hospital type, regions and cantons.

#### Availability of overview and detailed information

Since the information to be conveyed in a hospital comparison should be presented as clearly as possible, end users find it useful to divide the information provided into an overview and a detailed display. The overview display proposed in the new design solution was assessed by the end users as useful for recognising a potential quality problem at a glance. The ability to navigate to more detailed information about a hospital via the overview display is another interactive element that takes into account the diverse needs of end users. The additional detailed information provided is particularly valued by regulatory authorities, hospitals and professionals. For example, based on the historical data in the detailed display of the various quality indicators available for a hospital, their development over time can be estimated quickly and easily with just one click.

*I’m already doing the painstaking work of comparing the data from the hospitals over the years (…) Because the comparison is very important for me to see whether there are fluctuations that occur again and again or whether there is a decline or an improvement that is continuous and recognisable over several years. *[I-3]

In addition, the detailed presentation satisfies the demand for information on the risk-adjusted hospital effects and their 95% confidence intervals for the interested reader, without having to integrate these into the overview presentation (as in the caterpillar plot) for all end users.

### Suitability for quality development

Regardless of how clearly and interactively a hospital comparison is visualised, a further evaluation criterion is the extent to which the hospital comparison is perceived by the end users as suitable for stimulating quality improvement processes. In this context, two central criteria were identified as describing relevant conditions that favour/hinder the use of hospital comparisons for quality development.

#### Underlying data quality

For end users to consider a hospital performance comparison suitable for quality development, there must be confidence in the underlying data quality and the results need to be available in an appropriate time frame. Issues highlighted with respect to data quality included major uncertainties regarding the reliability of the results per hospital due to low case numbers and indications of possible misconduct on the part of hospitals when collecting data.

*So I’ve already heard from hospitals that (…) on the day they measure the falls (…) they call it the lying day*,* and then sometimes I think*,* oh okay*,* it’s really cute when it [the hospital performance comparison] looks nice*,* but they [hospitals] just cheat like crazy. *[I-4]

The other key criterion is how long it takes for the data and the hospital comparison to be available to end users and whether end users consider the timeliness of the results to be acceptable for quality development.

*Another point of criticism cannot be resolved in this way and that is the rapid availability of the results (…) until it’s available and so on*,* another year has passed and afterwards the hospitals rightly say (…) that was two years ago*,* in the meantime the situation is completely different.* [I-6]

#### Availability of reference values

The availability of reference values was highlighted by the end users as being important to inform quality development. As can be seen in Fig. 1, which is based on real data, there are only a few Swiss hospitals that deviate significantly negatively from the national average in terms of falls and pressure injuries after risk adjustment. In order to create additional incentives for continuous quality improvement activities, even in hospitals identified as inlier hospitals in the hospital comparison, it was suggested that the reference value used in the hospital comparison should be flexibly adjustable as required. For example, a comparison with the top 10% of hospitals as indicated by the following quote or with an international reference value could indicate potential for improvement.

*That would be a different approach*,* that you show the deviation from the top 10%*,* and show*,* hey*,* average is fine*,* but it would be better to be good.* [I-5]

### Quantitative evaluation

The link to the survey was sent to 611 targeted end users and clicked on by 426 of them (69.7%). Of these, 226 (53.1%) provided no or incomplete data. Therefore, 200 people completed the questionnaire in full (46.9% or 32.7% of the target population). The response rates in the two study groups did not differ significantly. In the control group 94 (44.5%) and in the intervention group 106 (49.3%) questionnaires were completed in full (Table 2). Neither did the two groups of participants who completed the questionnaire differ significantly in terms of contextual information.

**Table 2:** Descriptive and bivariate comparison of survey participants in the control and intervention groups

	Control (*n* = 211)		Intervention (*n* = 215)		
	*n*	%	*n*	%	*p*-value
Complete questionnaires	94	44.5	106	49.3	0.326
Swiss language region					
German	64	68.1	75	66.9	0.400
French/Bilingual	21	22.3	26	24.5	
Italian/Romansh	9	9.6	5	4.7	
Affiliation to hospital category					
University hospital	7	7.4	10	9.4	0.670
Centre hospital	35	37.2	32	30.2	
Regional hospital	27	28.7	37	34.9	
Specialised clinic	21	22.3	20	18.9	
Other organisation/Don’t know	4	4.3	7	6.6	
Approximate type of role in the organisation					
Hospital Director/Nursing Director	8	8.5	6	5.7	*0.364
(Head of) quality management	37	39.4	54	50.9	
Nursing expert	15	16.0	17	16.0	
Coordinator/site manager for NPM	33	35.1	29	27.4	
* Missing*	1	1.1	0	0.0	
Frequency of participation in the NPM					
Once	20	21.3	26	24.5	*0.491
Twice	3	3.2	7	6.6	
≥ 3 times	67	71.3	66	62.3	
Don’t know	4	4.3	7	6.6	

*Fisher’s exact test used, due to expected frequencies in cells < 5. NPM, National Prevalence Measurement of Falls and Pressure Ulcers

#### Correct interpretation

As Table 3 and Additional file 3 show, the number of outlier hospitals was correctly identified significantly more often in the intervention group than in the control group (correct identification of positive outliers *p* < 0.001 and correct identification of negative outliers *p* < 0.001). The example hospital in the hospital performance comparison, in contrast, was correctly identified as an inlier hospital in the two study groups with a similar frequency of around 70%. No difference was found between the groups (*p* = 0.161). Overall, the composite outcome indicates a better identification of deviating hospitals, as the frequency of correct interpretation with regard to outlier and inlier hospitals in the hospital performance comparison was significantly higher in the intervention group at 55.7%, compared with 12.8% in the control group (*p* < 0.001).

As the example hospital in the hospital comparison was shown as an inlier hospital in the graphs, the expected response from end users was that there was no need for action for that specific hospital. Here again, a significant group difference was found in that the end users in the control group (54.5%) compared with those in the intervention group (45.3%) more often correctly identified no need for action.

#### Perceived usability

End users in the intervention group reported a significantly higher average perceived usability (t(198) = 3.145, *p* = 0.002). The mean value of the SUS score in the intervention group was 10.5 (95% confidence interval 3.9–17.1) points higher than in the control group. At item level, slightly higher scores were achieved in the intervention group for all items. However, statistically significant group differences were only found for the items on ‘perceived complexity’, ‘perceived ease of use’ and ‘confidence in use’ (see Additional file 4).

**Table 3:** Comparison of the outcomes found in the control and intervention groups

	Control (*n* = 94)		Intervention (*n* = 106)		
Correct interpretation (outliers)	*n*	%	*n*	%	*p*-value
Correct identification of the number of positive outliers					
Yes	22	23.4	77	72.6	**< 0.001**
No	48	51.1	13	12.3	
Don’t know	24	25.5	16	15.1	
Correct identification of the number of negative outliers					
Yes	14	14.9	70	66.0	**< 0.001**
No	55	58.5	20	18.9	
Don’t know	25	26.6	16	15.1	
Correct identification of inlier (average) hospital					
Yes	66	70.2	79	74.5	0.161
No	21	22.3	14	13.2	
Don’t know	7	7.4	13	12.3	
**Composite outcome**	***n***	**%**	***n***	**%**	***p-value***
Correct identification of all positive, negative and inlier hospitals					
Yes	12	12.8	59	55.7	**< 0.001**
No	54	57.4	26	24.5	
Don’t know	28	29.8	21	19.8	
**Correct interpretation (need for action)**	***n***	**%**	***n***	**%**	***p-value***
Correct derivation of the need for action					
Yes	51	54.3	48	45.3	**0.039**
No	21	22.3	41	38.7	
Don’t know	22	23.4	17	16.0	
**Perceived usability**	Mean	SD	Mean	SD	***p-value***
SUS Score	42.8	21.9	53.3	24.9	**= 0.002**

Significant p-values are highlighted in bold. SUS, System Usability Scale; SD, standard deviation

## Discussion

The evaluation of a new design solution for the visualisation of hospital performance comparisons presented here showed that end users were highly positive about the new visualisation when comparing it to visualisation with caterpillar plots. Additionally, they reported understanding key messages better and perceived usability to be higher with the new design solution than with the caterpillar plot.

The criterion of clarity by design identified in our study largely confirms the statement ‘less is more’ by Peters et al. [26] in connection with the presentation of comparative quality information for consumers. The reduction of irrelevant design elements in the overview presentation of the new design solution – for example, the removal of confidence intervals and the ranking of hospitals – appeared to significantly improve the clarity of the information to be conveyed. This was also confirmed by the quantitative results of our study, as outlier hospitals were identified as such much more frequently. However, as an even higher percentage of correctly identified outlier hospitals (up to 81.8%) was achieved in another study [40], there is still room for improvement in this respect. It is expected that the clarity of the information conveyed by the overview display can be further improved for all end users by showing hospitals with only a coloured dot (reduction to a one-dimensional design element by omitting the historical value). In particular, it became clear in the interviews that the proposed two-dimensional design element (dot and surrounding circle) was difficult for end users to understand, at least without additional explanations. However, it also became clear in the end user interviews that although clarity is considered important, the reduction of additional information to achieve it is not equally welcomed by all end users. For example, the visualisation of uncertainties (e.g., with confidence intervals) is considered important by professionals (even if understanding is sometimes limited) [27, 32, 37], whilst the benefit for patients is questioned [23, 61]. This tension between the need for additional detailed information and the need to convey clear information through reduction and using familiar colour schemes, as described in our and other studies [19, 20, 27, 32], must be carefully weighed up in the design process. In order to meet the needs of all end users, this trade-off involves not only considering a reduction in the form of deleting information to improve clarity, but also separation or rearrangement of information in different displays, as proposed in the new design solution with an overview and one detailed display per hospital. The availability of various displays with different levels of information detail can make an important contribution to fulfilling the diverse needs of end users with regard to a hospital comparison [12, 17, 41, 62] and thus represents a central element of the usability by design criterion.

According to our study, usability by design also involves the introduction of end user-centred design elements that enable a certain degree of interactivity with the visualisation. Interactive design solutions that allow filtering, grouping and setting options to show and hide additional information seem well suited to meeting the diverse needs of end users [19, 20, 38]. Although the planned interactive functions of the design solution were considered appropriate and useful in the interviews, and the perceived usability was rated significantly better in direct comparison with the caterpillar plot in the quantitative evaluation, the still relatively low average SUS value of 53.3 (grade ‘D’) indicates considerable room for improvement. This potential is emphasised by the SUS value of 68 given by Lewis [57] as a reference median. Compared to this reference value, the low value found in our study could have been favoured by the fact that the evaluation was only based on a mock-up presentation, that is, on a static image in which the functionalities can only be visualised but not actually used. This approach required a certain amount of imagination for the end users to be able to picture, how the functionalities would be implemented in a final productive solution.

Interestingly, it became clear in the end user interviews that the suitability of a hospital comparison for decision making regarding quality improvement, another key criterion, not only depends on its visualisation. The suitability of hospital comparisons for quality improvement can generally be hampered by low expectations as to their benefits, as has been reported among hospital managers or healthcare professionals [13, 63–66]. Concerns about the underlying data quality can have a negative impact on these expectations. In the present study, for example, the end users questioned the reliability of the results in connection with the cross-sectional survey design and the associated low number of cases per hospital, a relevant aspect for the trustworthiness of performance comparisons [13]. Furthermore, absent or inadequate risk adjustment for differences in patient mix between hospitals may also lead to general distrust of any hospital comparison presented, as the comparability of hospitals may be called into question [40, 67]. Even when well-researched risk adjustment models underlie a hospital comparison, most end users are not familiar with the concept behind it and therefore do not understand exactly what it does; therefore concerns about the fairness of the comparison presented often remain [13, 25, 29, 46]. Finally, the time it takes for the results of a hospital comparison to become available to the end users, in particular service providers, has been shown to be a relevant inhibiting factor in the utilisation of the results for quality development in the event of a time delay [41, 63, 65, 68]. Using electronic medical routine data in the future, as proposed by Bernet et al. [49] for the indicators falls and pressure injuries in hospital, could significantly help to reduce end-user concerns about data quality and delayed availability of results.

In addition, a core function of hospital performance comparisons is to support accurate decisions about whether quality improvement actions are warranted [39, 40]. While a hospital identified as a negative outlier clearly indicates a need for action, the interpretation becomes less straightforward for hospitals performing within the national average range (i.e., inlier hospitals). This ambiguity is reflected in our findings: across both visualisations, approximately half of the participants correctly stated that no action was needed for an inlier hospital. Interestingly, participants in the control group, who viewed the current caterpillar plot, performed slightly better than those in the intervention group (54.5% vs. 45.3%).

One possible explanation for this finding is that the decision about whether action is required is more complex than simply identifying outlier status. End users may also take additional contextual information into account when drawing conclusions. Since more comprehensive contextual information is provided by the new design solution with the detailed display, such as trend data for multiple indicators, this additional information may have been taken into account by participants when deciding on the need for action.

### Strength and limitations

One of the strengths of this study is the interdisciplinary composition of the research core group and the involvement of a wide range of end users in developing and evaluating a new design solution for hospital performance comparison, an approach which is highly recommended in the literature [17, 40, 41]. It is important to note that, despite our efforts, patients were not represented in the study. The generalisability of the results for this group is therefore uncertain. It is thus imperative that this perspective is addressed in the further optimisation and testing of the new design solution.

Another strength is the mixed methods approach used. Firstly, we were able to gain a deeper understanding of the common key criteria that different end users rely on when evaluating a visualisation of a risk-adjusted hospital comparison. Secondly, we were able to test our hypotheses in a non-clinical randomised controlled trial (A/B testing) in a specific target group of end users with a substantial response rate at national level. The limitation that the quantitative results may not be representative of the entire range of potential end users must also be mentioned.

Another limitation to consider is that the number of hospitals displayed as negative outliers differed between the control group (*n* = 14) and the intervention group (*n* = 10). This adjustment was intended to counteract a learning effect in the undesirable case of a double entry. However, it also introduced unequal conditions between the groups, which may have influenced responses to this question, although the descriptive results do not suggest any such bias.

It should also be acknowledged that the differences between the current visualisation and the new design solution may not be due to the caterpillar plot format itself, but rather to its current implementation on the website. The current visualisation may lack key visualisation best practices, such as colour coding, a comprehensive legend, or explanatory text. It is therefore possible that the current visualisation with a caterpillar plot could also benefit from such enhancements. Future studies should consider using optimised versions of both visualisations to more accurately assess their respective strengths and limitations in conveying hospital performance comparisons.

## Conclusions

The new design solution for the visualisation of a risk-adjusted hospital comparison, developed and evaluated together with the end users, was rated positively in both qualitative and quantitative evaluations comparing it with the current visualisation using caterpillar plots. There is evidence that the new design solution, with its combination of more clearly understandable displays and interactive design elements, has the potential to improve the output of hospital comparisons for end users. It is recommended that the design solution, which is still at the mock-up stage, be converted to an interactive web-based prototype in order to test it more conclusively, particularly with regard to usability, and to trial it with end user groups that have not yet been reached, that is, patients or the interested general public. During further optimisation, it is also recommended that the enrichment with historical data in the basic setting of the overview display be omitted and instead possibly made available to end users as an additional setting. Furthermore, the colour scheme used in the new design should be evaluated using colour accessibility tools. Implementing customisable colour settings could improve usability for users with different needs. It is recommended that the key end user evaluation criteria identified in this study, namely, clarity by design, usability by design and suitability for quality development, be considered as relevant guiding criteria for reflection on and evaluation of future design projects addressing the visualisation of risk-adjusted hospital performance comparisons.

Simply measuring quality and providing the results does not guarantee that end users will use the information to inform their decisions. To increase the likelihood of uptake, the information should be easily accessible, understandable, timely, and relevant. Consequently, the new design solution focuses not only on aesthetics but also on promoting meaningful use, which is a prerequisite for public reporting to realise its potential as a strategy for quality improvement. In line with Berwick’s pathways, the newly proposed design solution enhances the ‘improvement through selection’ mechanism by enabling patients, insurers, and regulators to make informed, data-driven choices. It also strengthens the ‘improvement through change’ pathway by helping healthcare providers more easily identify and address quality issues. Thus, visualisation serves as a key intermediary between the availability of data and its practical application in decision-making and quality improvement. Ensuring that performance information is accessible, understandable, and actionable remains essential for public reporting to achieve its intended impact.

## Supplementary Information

Below is the link to the electronic supplementary material.

Supplementary Material 1

Supplementary Material 2

Supplementary Material 3

Supplementary Material 4

## Data Availability

The data on which the present results are based can be enquired upon reasonable request addressed to the corresponding author.

## Abbreviations

ANQ: Swiss National Association for Quality Development in Hospitals and Clinics
BFH: Bern University of Applied Sciences
NPM: National Prevalence Measurement of Falls and Pressure Ulcers
SUS: System Usability Scale
