# Supplementary material for: Evaluation of a new design solution for the visualisation of a risk-adjusted hospital performance comparison: results of an end user-centred mixed methods study
Source: BMC Med Inform Decis Mak. 2026 Apr 22;26:207. doi: 10.1186/s12911-026-03501-5 (PMC13235198; doi:10.1186/s12911-026-03501-5)
Supplement: Supplementary file 1 — Supplementary Material 1 [file 12911_2026_3501_MOESM1_ESM.pdf]

Additional file 1

Phase 1: Needs assessment and problem identification

In the first phase, explorative qualitative methods were used to investigate end users' experiences with the current visualisation approach and their expectations/needs for a new design solution of risk-adjusted hospital performance comparisons. The relevant data was collected by means of a focus group. In addition, an online survey with open questions was distributed to the end users participating in the study as a preparatory task for the focus group.

End user recruitment

We applied a purposive sampling strategy for the recruitment of end users [1], as information needs among end users may differ considerably [2]. The purposive sampling strategy was therefore designed to ensure the inclusion of relevant end users from different levels of the healthcare system in the study. Based on literature [2, 3] and in dialogue with the National Association for Quality Development in Hospitals and Clinics (ANQ) as the stakeholder gatekeeper, we first identified relevant entities whose representatives could be assumed to work with risk-adjusted hospital comparisons or use them for decision-making purposes and therefore be potential end users of a new visualisation design solution. We aimed to recruit at least one representative from the following entities: national government (Federal Office of Public Health and Federal Quality Commission), cantonal government (cantonal health departments), health insurers, hospital association H+, ANQ, hospitals and, as potential patients, the general public. Through personal contacts in the networks of the research group and the ANQ, potential representatives of the defined entities were contacted and informed about the research project at a personal meeting to clarify whether they could be formally asked by the core research group to take part in the study. The potential participants were then sent the written study information by email and officially invited by the research group to take part in the study. Initially, a representative was recruited from all the entities approached. However, during the recruitment process, the patient withdrew for unknown reasons. Ultimately, a total of 16 representatives from 11 entities took part in the study. One representative from the Federal Office of Public Health as well as from the Federal Quality Commission, two from two cantonal health departments, one from health insurers, one from the hospital association H+, one from ANQ and nine from four hospitals (nursing experts and managers, IT specialists). No second recruitment was carried out due to a tight time schedule.

Preparatory online survey

As a preparatory task for the focus group discussion, an online survey was conducted with the participating end users in February 2021. The survey contained a total of nine open-ended questions defined by the core research team and implemented in SurveyMonkey (www.surveymonkey.com). The link to the online survey was sent to the end users by email together with the invitation and programme for the subsequent focus group discussion. The representatives were asked to complete at least one questionnaire per entity if possible. The survey was conducted anonymously.

The preparatory survey aimed to engage the end users' minds with the subject of the focus group and to gather an initial impression of the current state and future expectations of national quality measurements and the results derived, focussing on their meaning, communication and interpretation challenges. The survey was divided into three main topic areas, with each part being introduced before the corresponding questions (see Table 1, Additional file 1 for more details). Part 1 focuses on end users' views on the importance, use and desired presentation of results of national quality measurements. Part 2 focuses on the challenges and support needed to interpret risk-adjusted results of national quality measurements. Part 3 collects information on potential additional end users and topics of interest related to national quality measurements and the presentation of results. We received a total of 9 completed surveys, which were then summarised by the core research team.

Table 1, Additional file 1: Overview of the structure and questions of the preparatory online survey

**Part 1 - Results of national quality measurements: expectations, purpose and visualisation**	
1.	Why are national quality measurements important to you and how have you used the results so far, if at all?
2.	What benefits do you hope to gain from national quality measurements in the future?
3.	What data/key figures need to be collected and visualised so that you can use the results of national quality measurements in line with your expectations?
4.	In what form are the results of (national) quality measurements currently communicated? (Examples: brochures, FAQs, dashboards, reports, etc.)<br>- Formats used?<br>- Which of these works well for communicating results?<br>- What is currently missing for successful communication of results? What would you like to see?
5.	Assuming that all results are communicated digitally (website, app, dashboards, etc.), what functions should this digital solution include? Which views of the results (data) should be provided so that you can use them according to your expectations?
**Part 2 - Risk adjustment**	
6.	If you have already dealt with risk-adjusted results, what do you think makes the interpretation of these results difficult?
7.	How could the interpretation of results be supported?
**Part 3 - Final questions regarding end users and other topics**	
8.	In your view, are there other stakeholders or interest groups for national quality measurements?
9.	In your opinion, are there any additional specific topics for the workshop?

Focus group discussion

The summarised findings of the preparatory survey were used to structure the focus group discussion in order to elaborate and validate the results obtained in discussion with the end users. In this context, the focus group discussion emphasised the joint development of possible user scenarios. To this end, all participants were asked to sketch possible user scenarios on a digital pinboard (padlet). The user scenarios were grouped ad hoc and possible practical implications for the design process were collected, discussed and defined together. Therefore, the main objective of the focus group discussion was to create a common ground (between the end users and the specialists) with regard to expectations and needs in relation to national quality measurements and the corresponding presentation of results in

order to guide the further course of the project and in particular the design of how the (risk-adjusted) results are presented. The focus group with end users took place online (due to the COVID-19 pandemic) via MS Teams in March 2021 and lasted around two hours. The focus group was video-recorded with participant consent obtained before the start of the video recording. The main findings from the focus group discussion were summarised by the core research team in the form of a short report and was sent to the end users for a member check [4].

Main results of phase 1 regarding a new design solution

It was confirmed that the requirements for national quality measurements and the abilities of the various end users to utilise the generated data (hospital comparisons) are highly heterogeneous. The end users and core research group agreed that the national risk-adjusted hospital comparison is a cornerstone of quality measurement and that its visualisation is essential in a new design solution. Additionally, it was emphasised that a digital solution is needed that is as flexible as possible (e.g., expandable to several quality indicators based on different national quality measurements). On the basis of the jointly developed data usage scenarios, it became clear that the simplest possible and immediately interpretable forms of presentation that lead to an ‘aha effect’ regardless of prior knowledge through to detailed views that can be adapted to the individual needs of the end user (keyword: filter options or grouping options) were required. The need for capability to map quality over time so that, for example, it is possible to intuitively monitor whether a particular hospital has improved/developed also emerged. Finally, the new design solution should also help end users to make better use of the data, for example, by making it easier for non-experts to understand the processes behind risk adjustment and its significance in a comparison.

Phase 2: Development of a new design solution

Building on the findings from phase 1 and incorporating systematic literature reviews [5-7], an iterative design process was launched in summer 2021 to develop a new design solution. Initially, various solution approaches were outlined under the leadership of the design expert as part of an ideation process. These were discussed within the core research group, then continuously adapted and further developed in an iterative process. During the development process it emerged that a visualisation with different gradations, from the simplest possible views that can be interpreted independently of prior knowledge to detailed views according to individual needs, seemed most appropriate. The new design solution attempted to promote intuitive readability through new information architecture and visual reduction to the relevant information. Specifically, an overview display was created in which 1) all visual information important from a scientific point of view but of little relevance for the interpretation of the results (e.g., confidence intervals) was removed, 2) the proposed ranking list was replaced by a matrix, 3) options for clustering and comparisons over time were created. Additionally, a new detailed display was created that allows an extended interpretation of the available data per hospital (adjusted vs. unadjusted results, risk adjusters, historical trend data). The proposed core elements of the new design solution were visualised in a static mock-up presentation in order to discuss and evaluate the new design solution with the end users in phase 3.

References

1. Martínez-Mesa, J., et al., *Sampling: how to select participants in my research study?* Anais Brasileiros de Dermatologia, 2016. **91**(3): p. 326-330. https://doi.org/10.1590/abd1806-4841.20165254
2. Busse, R., et al., *Improving healthcare quality in Europe: Characteristics, effectiveness and implementation of different strategies*, in *Health Policy Series*. 2019, World Health Organization, Regional Office for Europe and Organisation for Economic Co-operation and Development: Copenhagen. https://iris.who.int/handle/10665/327356
3. Smith, P.C., et al., *Performance Measurement for Health System Improvement: Experiences, Challenges and Prospects*. Health Economics, Policy and Management. 2010, Cambridge: Cambridge University Press.
4. Birt, L., et al., *Member Checking: A Tool to Enhance Trustworthiness or Merely a Nod to Validation?* Qualitative Health Research, 2016. **26**(13): p. 1802-1811. https://doi.org/10.1177/1049732316654870
5. Hancock, S.L., et al., *Feedback of patient-reported outcomes to healthcare professionals for comparing health service performance: a scoping review.* BMJ Open, 2020. **10**(11): p. 1-25. https://doi.org/10.1136/bmjopen-2020-038190
6. Kurtzman, E.T. and Greene, J., *Effective presentation of health care performance information for consumer decision making: A systematic review.* Patient Education and Counseling, 2016. **99**(1): p. 36-43. https://doi.org/10.1016/j.pec.2015.07.030
7. Peters, E., et al., *Less Is More in Presenting Quality Information to Consumers.* Medical Care Research and Review, 2007. **64**(2): p. 169-190. https://doi.org/10.1177/10775587070640020301
