# Supplementary material for: Evaluation of a new design solution for the visualisation of a risk-adjusted hospital performance comparison: results of an end user-centred mixed methods study
Source: BMC Med Inform Decis Mak. 2026 Apr 22;26:207. doi: 10.1186/s12911-026-03501-5 (PMC13235198; doi:10.1186/s12911-026-03501-5)
Supplement: Supplementary file 2 — Supplementary Material 2 [file 12911_2026_3501_MOESM2_ESM.pdf]

Additional file 2

Development and translation of the questionnaire for the quantitative analysis

The questionnaire used in the survey was compiled by the core research team and can be divided into three parts: a general part ‘A’ with four contextual questions about the end user (e.g., function at work or how often they had already been involved in carrying out the National Prevalence Measurement of Falls and Pressure Ulcers (NPM)); part ‘B’ to check the correct interpretation of the visual information presented; part ‘C’ to assess the usability of the hospital performance comparison presented (see Table 1, Additional file 2 for a complete overview).

For part ‘B’, correct interpretation was understood as ‘*the ability to derive correct conclusions about who is performing well and who is not [...] Correct interpretation is often seen as a key ability to use information correctly*’ [1]. To assess how well the visualisation conveys key messages, we formulated four questions based on similar research [1-5]. The first two questions were aimed at determining whether there were hospitals in the hospital comparison that deviated, first, positively and, second, negatively from the total number of hospitals, and if so, how many. As it was technically possible for a participant to complete the survey twice because of anonymity, we slightly modified the design solution in comparison with the current visualisation with respect to one question in the survey (i.e., the number of hospitals with negative deviations (=14 [control]/10 [intervention]) to counteract a learning effect in the undesirable case of a double entry.

The other two questions were used to check how well the visualisation could reflect the average of the corresponding hospitals by asking how the example hospital in the graph performed in comparison with the total number of hospitals and whether quality improvement measures should be taken on the basis of this result.

In part ‘C’, respondents' perception of the usability of the hospital performance comparison presented was assessed using the System Usability Scale (SUS) originally developed by John Brook in 1986 [6]. The SUS is a widely used, psychometrically tested instrument for determining perceived usability based on 10 items, with a 5-point Likert scale from ‘strongly disagree’ to ‘strongly agree’ [7, 8]. If a question could not be answered, the participants were asked to select the middle (3) answer category in accordance with the SUS instructions. In order to adapt the SUS to the context of our study, we used the modified SUS of Bangor, Kortum, et al. [7] as the English reference version. Specifically, we replaced the terms ‘product’ with ‘hospital comparison’ and changed the tense from past to present where necessary, as our hospital performance comparisons were only available as mock-up images and not in an effectively usable environment. In order to have an SUS that is as harmonised as possible for use at national level in multilingual Switzerland, we have reconciled the versions available in German [9, 10], French [9, 11] and Italian [12] with each other and with the English reference version [7].

Overall, the complete questionnaire and all necessary adaptations in the SUS with regard to the individual languages were carried out according to Wild, Grove, et al. [13] in a forward and backward translation process by two persons familiar with the respective languages. The questionnaire also provided the opportunity to comment on the information provided in text fields.

Table 1, Additional file 2: Description of the questionnaire used

**Part A: contextual questions about the end users**	
1.	What type of hospital do you work in?
2.	In which language region do you work?
3.	Please indicate in which function you work in your hospital.
4.	Please indicate how often you have participated in the NPM.
**Part B: questions to assess 'correct interpretation' of the visual information presented**	
6.	Are there any hospitals in the hospital comparison that deviate positively from the total of Swiss hospitals?
	- If so, how many?
7.	Are there any hospitals in the hospital comparison that deviate negatively from the total of Swiss hospitals?
	- If so, how many?
8.	How does the example hospital in the graph compare to the total of Swiss hospitals?
9.	Would you take quality improvement measures based on the results of the example hospital?
**Part C: System Usability Scale (SUS)**	
10.	I think that I would like to use this hospital comparison frequently.
11.	I found the hospital comparison unnecessarily complex.
12.	I thought this hospital comparison was easy to use.
13.	I think that I would need the support of a technical person to be able to use this hospital comparison.
14.	I found the various functions in this hospital comparison were well integrated.
15.	I thought there was too much inconsistency in this hospital comparison.
16.	I would imagine that most people would learn to use this hospital comparison very quickly.
17.	I found this hospital comparison very awkward to use.
18.	I felt very confident using this hospital comparison.
19.	I needed to learn a lot of things before I could get going with this hospital comparison.

References

1. Damman, O.C., et al., *Consumers' interpretation and use of comparative information on the quality of health care: the effect of presentation approaches.* Health Expectations, 2012. **15**(2): p. 197-211. https://doi.org/10.1111/j.1369-7625.2011.00671.x
2. Geraedts, M., Hermeling, P., and de Cruppé, W., *Communicating quality of care information to physicians: A study of eight presentation formats.* Patient Education and Counseling, 2012. **87**(3): p. 375-382. https://doi.org/10.1016/j.pec.2011.11.005
3. Damman, O.C., et al., *Making comparative performance information more comprehensible: an experimental evaluation of the impact of formats on consumer understanding.* BMJ Quality & Safety, 2016. **25**(11): p. 860-869. https://doi.org/10.1136/bmjqs-2015-004120
4. Marshall, T., Mohammed, M.A., and Rouse, A., *A randomized controlled trial of league tables and control charts as aids to health service decision-making.* International Journal for Quality in Health Care, 2004. **16**(4): p. 309-315. https://doi.org/10.1093/intqhc/mzh054
5. Peters, E., et al., *Less Is More in Presenting Quality Information to Consumers.* Medical Care Research and Review, 2007. **64**(2): p. 169-190. https://doi.org/10.1177/10775587070640020301
6. Brooke, J.B., *SUS: A 'Quick and Dirty' Usability Scale*, in *Usability Evaluation In Industry*, P.W. Jordan, et al., Editors. 1996, CRC Press: London. https://doi.org/10.1201/9781498710411
7. Bangor, A., Kortum, P.T., and Miller, J.T., *An Empirical Evaluation of the System Usability Scale.* International Journal of Human–Computer Interaction, 2008. **24**(6): p. 574-594. https://doi.org/10.1080/10447310802205776
8. Lewis, J.R., *The System Usability Scale: Past, Present, and Future.* International Journal of Human–Computer Interaction, 2018. **34**(7): p. 577-590. https://doi.org/10.1080/10447318.2018.1455307
9. Gao, M., Kortum, P., and Oswald, F.L., *Multi-Language Toolkit for the System Usability Scale.* International Journal of Human–Computer Interaction, 2020. **36**(20): p. 1883-1901. https://doi.org/10.1080/10447318.2020.1801173
10. Rummel, B. *System usability scale–jetzt auch auf deutsch*. 2016 [cited 10.11.2021]. Available from: https://community.sap.com/t5/additional-blogs-by-sap/system-usability-scale-jetzt-auch-auf-deutsch/ba-p/13487686
11. Gronier, G. and Baudet, A., *Psychometric Evaluation of the F-SUS: Creation and Validation of the French Version of the System Usability Scale.* International Journal of Human–Computer Interaction, 2021. **37**(16): p. 1571-1582. https://doi.org/10.1080/10447318.2021.1898828
12. Borsci, S., Federici, S., and Lauriola, M., *On the dimensionality of the System Usability Scale: a test of alternative measurement models.* Cognitive Processing, 2009. **10**(3): p. 193-197. https://doi.org/10.1007/s10339-009-0268-9
13. Wild, D., et al., *Principles of Good Practice for the Translation and Cultural Adaptation Process for Patient-Reported Outcomes (PRO) Measures: Report of the ISPOR Task Force for*

Translation and Cultural Adaptation. Value in Health, 2005. **8**(2): p. 94-104.
https://doi.org/10.1111/j.1524-4733.2005.04054.x
