# Supplementary material for: Evaluation of a new design solution for the visualisation of a risk-adjusted hospital performance comparison: results of an end user-centred mixed methods study
Source: BMC Med Inform Decis Mak. 2026 Apr 22;26:207. doi: 10.1186/s12911-026-03501-5 (PMC13235198; doi:10.1186/s12911-026-03501-5)
Supplement: Supplementary file 3 — Supplementary Material 3 [file 12911_2026_3501_MOESM3_ESM.pdf]

Additional file 3

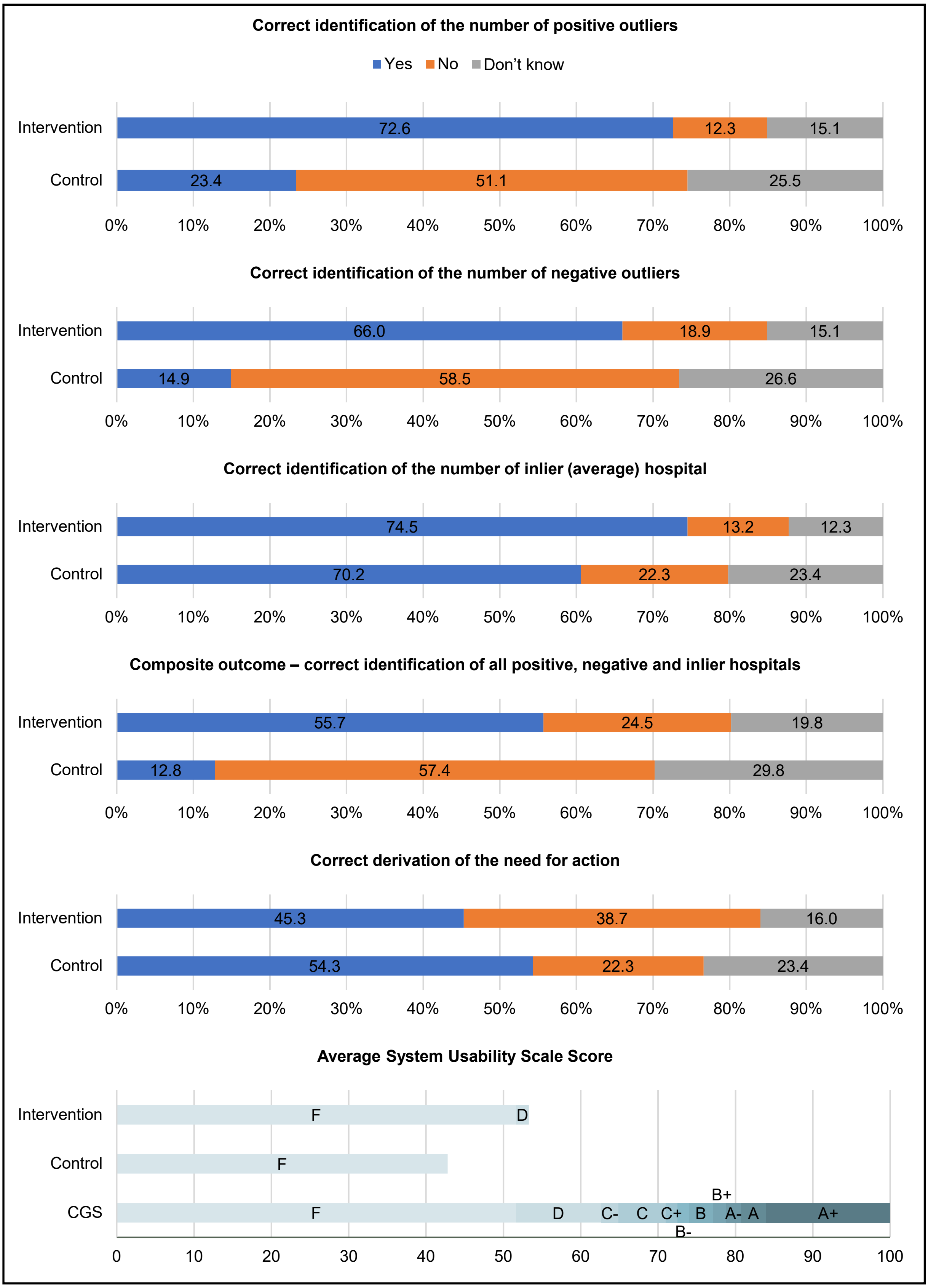

Figure 1, Additional file 3: Graphical comparison of the outcomes in the control and intervention groups. CGS, curved grading scale according to Sauro and Lewis [1].

References

1. Sauro, J. and Lewis, J.R., *Quantifying the user experience practical statistics for user research*. 2012, Waltham, Massachusetts: Morgan Kaufmann.
