# Supplementary material for: Evaluation of a new design solution for the visualisation of a risk-adjusted hospital performance comparison: results of an end user-centred mixed methods study
Source: BMC Med Inform Decis Mak. 2026 Apr 22;26:207. doi: 10.1186/s12911-026-03501-5 (PMC13235198; doi:10.1186/s12911-026-03501-5)
Supplement: Supplementary file 4 — Supplementary Material 4 [file 12911_2026_3501_MOESM4_ESM.pdf]

Additional file 4

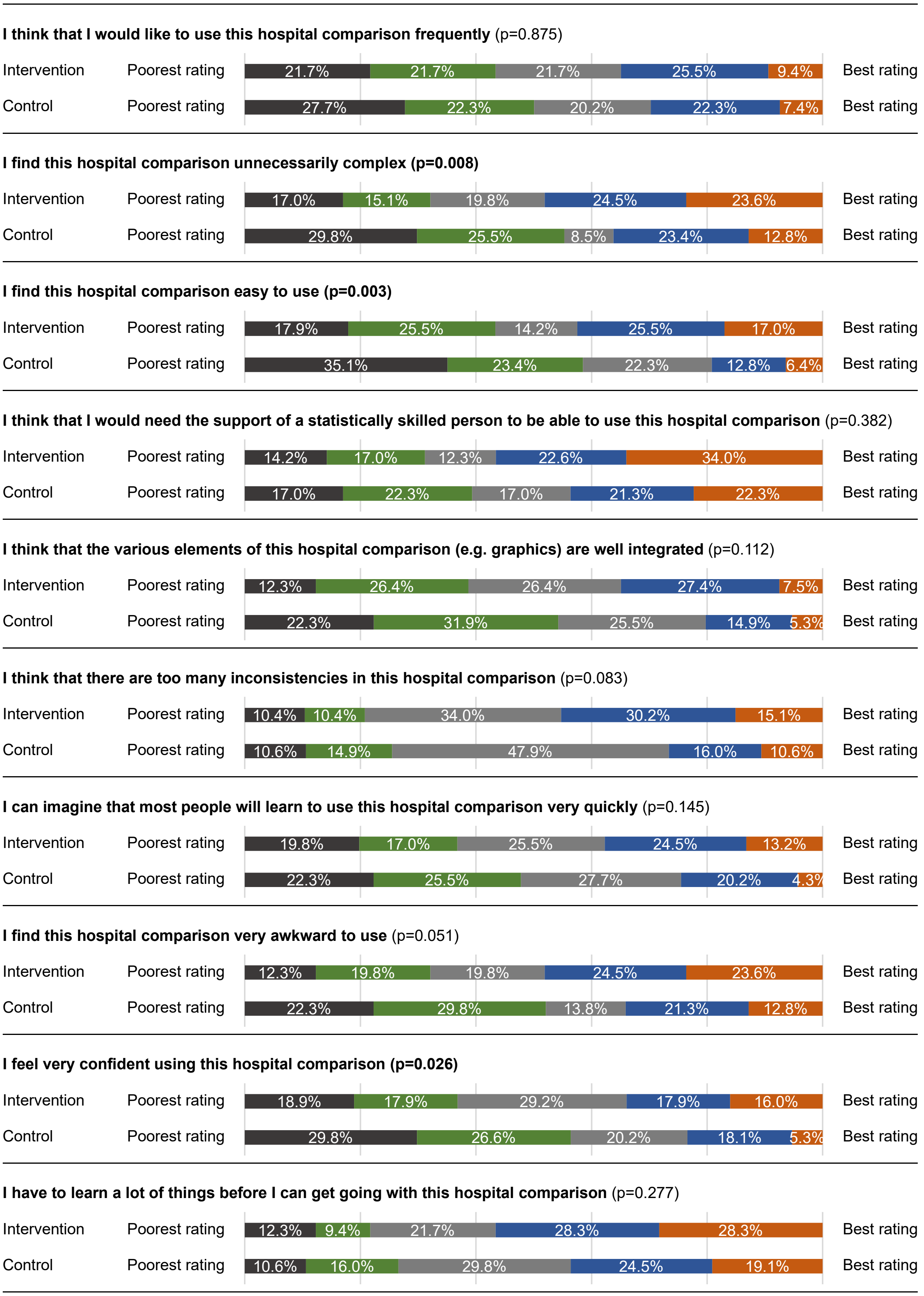

Figure 1, Additional file 4: Comparison of the ratings of the System Usability Scale items in the intervention and control groups. Significant differences are highlighted in bold.
